# Downregulation of MicroRNA-1 and Its Potential Molecular Mechanism in Nasopharyngeal Cancer: An Investigation Combined with In Silico and In-House Immunohistochemistry Validation

**DOI:** 10.1155/2022/7962220

**Published:** 2022-02-25

**Authors:** Jia-Ying Wen, Li-Ting Qin, Gang Chen, He-Qing Huang, Ming-Jun Shen, Jin-Shu Pang, Yu-Xing Tang, Wei Lu, Ren-Sheng Wang, Jia-Yuan Luo

**Affiliations:** ^1^Department of Radiotherapy, First Affiliated Hospital of Guangxi Medical University, Nanning, Guangxi Zhuang Autonomous Region, China; ^2^Department of Pathology, First Affiliated Hospital of Guangxi Medical University, Nanning, Guangxi Zhuang Autonomous Region, China; ^3^Department of Pathology, Nanning Second People's Hospital, Third Affiliated Hospital of Guangxi Medical University, Nanning, Guangxi Zhuang Autonomous Region, China

## Abstract

**Background:**

This study was aimed at elucidating the molecular biological mechanisms of microRNA-1 (miR-1) in nasopharyngeal carcinoma (NPC).

**Method:**

In this study, we performed a pooled analysis of miR-1 expression data derived from public databases, such as GEO, ArrayExpress, TCGA, and GTEx. The miRWalk 2.0 database, combined with the mRNA microarray datasets, was used to screen the target genes, and the genes were then subjected to Kyoto Encyclopedia of Genes and Genomes (KEGG) and Gene Ontology (GO) enrichment analysis using the DAVID 6.8 database. We then used the STRING 11.0 database and Cytoscape 3.80 software to construct a protein-protein interaction (PPI) network for screening hub genes. Immunohistochemistry (IHC) was further used to validate the expression of hub genes. Finally, potential therapeutic agents for NPC were screened by the Connectivity Map (cMap) database.

**Results:**

Pooled analysis showed that miR-1 expression was significantly decreased in NPC (SMD = −0.57; *P* < 0.05). The summary receiver operating characteristic curve suggested that miR-1 had a good ability to distinguish cancerous tissues from noncancerous tissues (AUC = 0.78). The results of GO analysis focused on mitotic nuclear division, DNA replication, cell division, cell adhesion, extracellular space, kinesin complex, and extracellular matrix (ECM) structural constituent. The KEGG analysis suggested that the target genes played a role in key signaling pathways, such as cell cycle, focal adhesion, cytokine-cytokine receptor interaction, ECM-receptor interaction, and PI3K/Akt signaling pathway. The PPI network suggested that cyclin-dependent kinase 1 (CDK1) was the hub gene, and the CDK1 protein was subsequently confirmed to be significantly upregulated in NPC tissues by IHC. Finally, potential therapeutic drugs, such as masitinib, were obtained by the cMap database.

**Conclusion:**

miR-1 may play a vital part in NPC tumorigenesis and progression by regulating focal adhesion kinase to participate in cell mitosis, regulating ECM degradation, and affecting the PI3K/Akt signaling pathway. miR-1 has the potential to be a therapeutic target for NPC.

## 1. Introduction

Nasopharyngeal carcinoma (NPC) is a malignant tumor arising from the nasopharyngeal epithelium [1]. NPC is a highly regionally related tumor. The incidence of NPC is high in many provinces in southern China, Southeast Asian countries, northern and northeastern Africa, Alaska in the United States, western Canada, and so on [2–8]. According to epidemiological statistics, the incidence of NPC in southern China is remarkably higher than the average in the world [9–11]. NPC is sensitive to radiotherapy, and with the development of radiotherapy technology, the five-year survival rate of early NPC is as high as 95%. However, due to the insidious location of NPC and the difficulty in distinguishing NPC from benign diseases by symptoms, most patients with NPC are diagnosed at a late stage, with neck lymph nodes and/or distant metastasis. Therefore, elucidating the pathogenesis of NPC and determining new biomarkers and therapeutic targets are urgent.

MicroRNAs (miRNAs) are a class of small noncoding endogenous RNAs, with a length of approximately 21 to 23 nucleotides [12–15]. Aberrant miRNA expression has been associated with multiple pernicious tumors in humans, reflecting their critical biological roles. miR-1 expression is downregulated in various human tumors, including osteosarcoma, bladder cancer, and gastric cancer [16–18]. Wu et al. confirmed that miR-1 expression was downregulated in colorectal cancer, and the overexpression of miR-1 enhanced the radiotherapy sensitivity of colorectal cancer by inducing apoptosis and ultimately acted as a tumor suppressor [19]. Our previous study also demonstrated that the expression of miR-1 was significantly downregulated in both prostate cancer and clear cell renal cell carcinoma, and miR-1 affected the tumorigenesis and invasion of the above tumors by regulating the expression of the target genes [20, 21]. Besides, Jin et al. found that MALAT1 can affect cancer stem cell activity and NPC radioresistance by regulating the miR-1/slug axis [9].

To further investigate the expression and targeted therapeutic value of miR-1 in NPC, we performed immunohistochemistry (IHC) and a comprehensive analysis, including data from public databases. This analysis revealed that miR-1 is downregulated in NPC and has the potential to be a new therapeutic target.

## 2. Materials and Methods

### 2.1. Collecting Microarray Datasets

All mRNA and miRNA microarray datasets included in this study were derived from databases, such as GEO, ArrayExpress, TCGA, and GTEx. Key search terms were ((*mRNA* OR *messageRNA*) AND (*tumor* OR *cancer* OR *tumour* OR *carcinoma* OR *neoplas*∗ OR *malignan*∗) AND (*nasopharynx* OR *nasopharyngeal* OR *rhinopharyngeal* OR *NPC*)) to obtain the mRNA microarray datasets. The retrieval formula of miRNA microarray datasets was ((*miRNA* OR *microRNA*) AND (*cancer* OR *malignan*∗ OR *neoplas*∗ OR *tumor* OR *carcinoma*) AND (*nasopharynx* OR *nasopharyngeal* OR *rhinopharyngeal* OR *NPC*)). The deadline for literature retrieval was October 2020. Each microarray dataset in this study must include an NPC group and a noncancer group. The exclusion criteria for the data were as follows: (1) animal-based studies were eliminated and (2) studies with less than three samples were culled. The processing of microarray datasets followed the following principles: (1) expression data without normalization processing was log2 transformed and (2) missing values of the datasets were replaced with results calculated by *k*-nearest neighbor (KNN).

### 2.2. Analyzing miR-1 Expression in NPC Tissues

All included miRNA microarray datasets were summarized and analyzed using Stata 16.0, and the standardized mean difference (SMD) was calculated to obtain reliable results. miR-1 expression was relatively low in cancer tissues if the SMD was <0 and if the 95% confidence interval (CI) did not cross the 0-point coordinate line; by contrast, miR-1 expression was high in cancer tissues. Sensitivity analysis was then performed to measure the reliability and stability of the pooled analysis results, and the publication bias was detected by Begg's method. The threshold for statistical significance was *P* < 0.05.

In addition, to measure the ability of miR-1 to discriminate between cancerous and noncancerous tissues, we calculated the receiver operating characteristic (ROC) curve of individual datasets and the summary ROC (SROC) curve of all datasets and the area under the curve (AUC), respectively.

### 2.3. Searching for Potential Target Genes of miR-1 in NPC Tissues

miRWalk is an integrative database that includes the information of miRNA target genes in humans, mice, and other species and integrates the data from miRBridge, miRDB, miRanda, TargetScan, and other databases.

In this study, 12 databases, including miRWalk, miRDB, miRBridge, and other databases in miRWalk2.0, were applied to obtain the target genes of miR-1. Meanwhile, differentially expressed genes (DEG) in the mRNA datasets of NPC derived from the public databases were also screened using the limma package of the R x64 3.6.2 software. Genes with a |log2 (fold change)| of >1 and *P* < 0.05 were considered DEGs. Intersection was then obtained for all upregulated DEGs, with genes present in at least two sets as the candidate target genes. Finally, the selected upregulated DEGs were intersected with the genes predicted by miRWalk2.0 to obtain target genes for downstream analysis.

### 2.4. Analyzing Target Gene Function by Gene Ontology (GO) and Kyoto Encyclopedia of Genes and Genomes (KEGG)

GO is a database that can identify the gene and protein functions of various species and elucidate the biological functions involved in target genes from three aspects: biological process (BP), molecular function (MF), and cellular component (CC).

KEGG is an integrative database that includes chemical, genomic, and system functional data. It can identify the vital signal transduction pathways and biochemical metabolic pathways of target genes.

In this study, GO analysis and KEGG signaling pathway analysis were conducted by the DAVID 6.8 database to explore the biological functions and cellular signaling pathways of miR-1 during NPC tumorigenesis and progression.

### 2.5. Constructing the Protein-Protein Interaction (PPI) Network

STRING is a database that searches for interactions between proteins, which includes 2,031 species, 9.6 million proteins, and 13.8 million interaction relationships between proteins. Cytoscape 3.80 is bioinformatic analysis software for constructing molecular interaction network diagrams. Each node of the diagram represents a gene, protein, or molecule, and the lines between nodes indicate interaction.

We constructed a miR-1 target gene PPI network (high confidence > 0.70) using the STRING 11.0 database and calculated the degree number by the Centiscape2.2 plugin of Cytoscape [22–24]. In the PPI network, the most highly connected genes were identified as hub genes.

### 2.6. Validating Target Gene Expression by In-House IHC

Thirty-six NPC tissues and twenty-eight chronic inflammatory tissues of the nasopharynx mucosa collected from the First Affiliated Hospital of Guangxi Medical University were subjected to IHC staining to detect the expression of target genes. Two experienced pathologists assessed the differences in stained areas by the immunoreactivity score (IRS).

Ten typical high magnification fields were randomly observed under an optical microscope. The IRS was calculated from the staining intensity and the percentage of stained cells in each sample. The scores of staining intensity were classified into four grades: 0, 1, 2, and 3, representing none, weak, medium, and strong staining, respectively. The staining range was divided into five grades. For example, NPC tissues without staining were recorded as 0, <25% stained cells were recorded as 1, 25%–49% stained cells were recorded as 2, 50%–74% stained cells were recorded as 3, and more than 74% stained cells were recorded as 4. The scores of staining intensity and staining range were subsequently multiplied to generate IRS [25].

Finally, the IRS of NPC tissues and chronic inflammatory tissues of the pharyngeal mucosa was compared using an independent sample *t*-test using SPSS 26.0. The threshold for statistical significance was *P* < 0.05. The Ethics Committee of the First Affiliated Hospital of Guangxi Medical University approved the above experiments.

### 2.7. Screening Candidate Therapeutic Agents

The Connectivity Map (cMap) is an online database for exploring the relationship between molecule drugs, gene expressions, and diseases [26–29]. The above upregulated and downregulated DEGs screened by the limma package were entered into the cMap database and compared with the reference datasets. The degree of similarity is assessed by score. The value range of the score is between −100 and 100. The closer the value is to 100, the more similar the gene list is to this small-molecule processing record. The closer the value is to −100, the more opposite the gene list is to this small-molecule processing record, suggesting that this small-molecule compound exhibits an antagonistic effect on NPC and has the potential to be a targeted therapeutic agent for NPC.

## 3. Results

### 3.1. Expression of miR-1 in NPC

**Figure 1:**
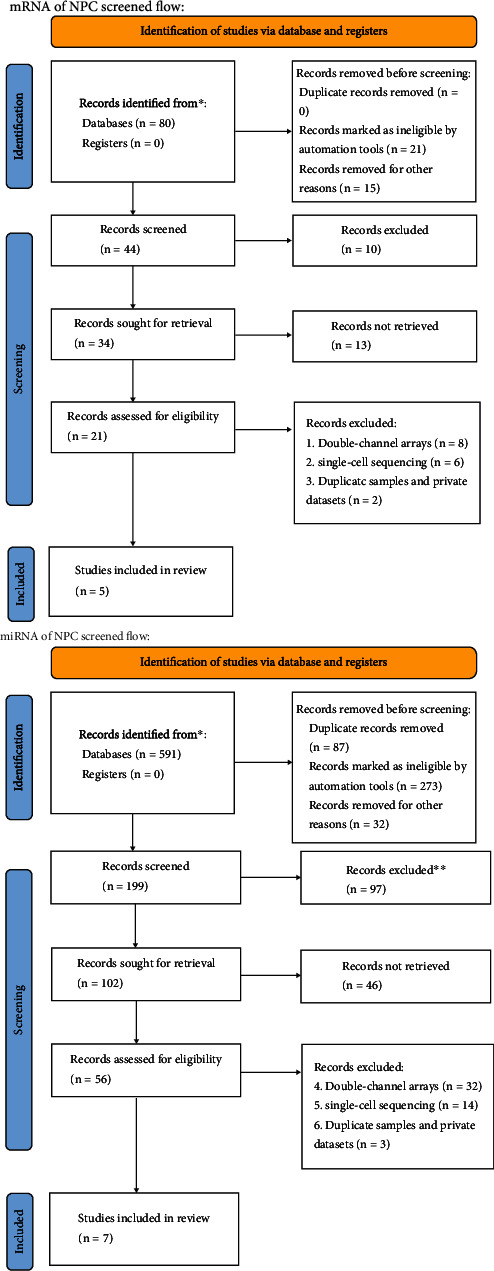
The process of screening NPC-related datasets from public databases. After screening, a total of five mRNA microarray datasets and seven miRNA microarray datasets of NPC were obtained.

**Figure 2:**
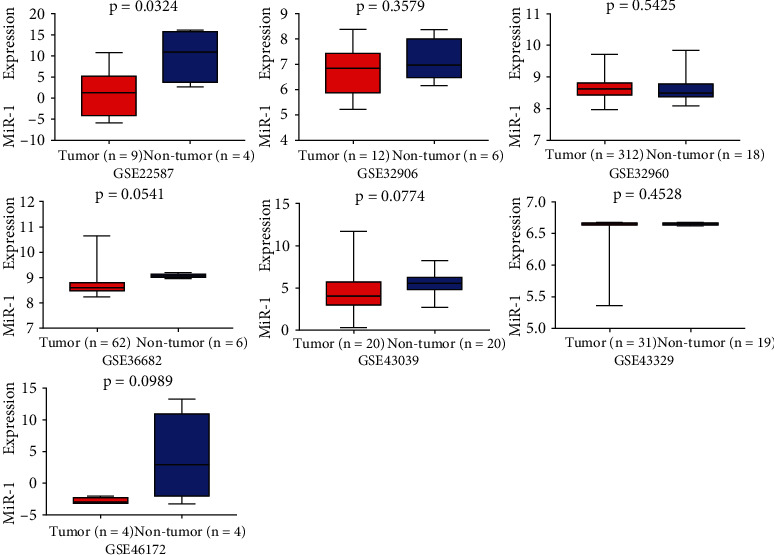
Expression of miR-1 in NPC and noncancerous microarray datasets.

According to the screening process shown in Figure 1, in this study, seven miRNA microarray datasets (GSE22587, GSE32906, GSE32960, GSE36682, GSE43329, GSE43039, and GSE46172) and five mRNA microarray datasets (GSE12452, GSE13597, GSE34573, GSE53819, and GSE64634) were selected from databases, such as GEO, ArrayExpress, TCGA, and GTEx.  Figure 2 visualizes the expression of miR-1 in the individual microarray datasets, which shows that miR-1 in some microarray datasets is relatively downexpressed in NPC.

**Figure 3:**
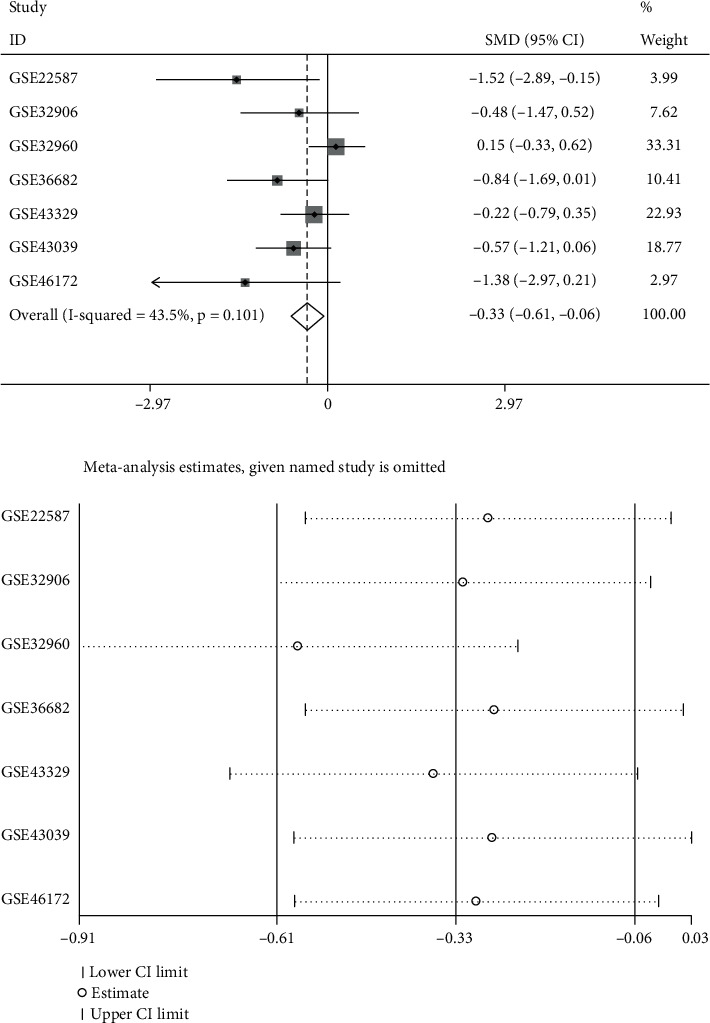
Pooled expression of miR-1 was calculated using a random effects model: (a) forest plot and (b) sensitivity analysis.

**Figure 4:**
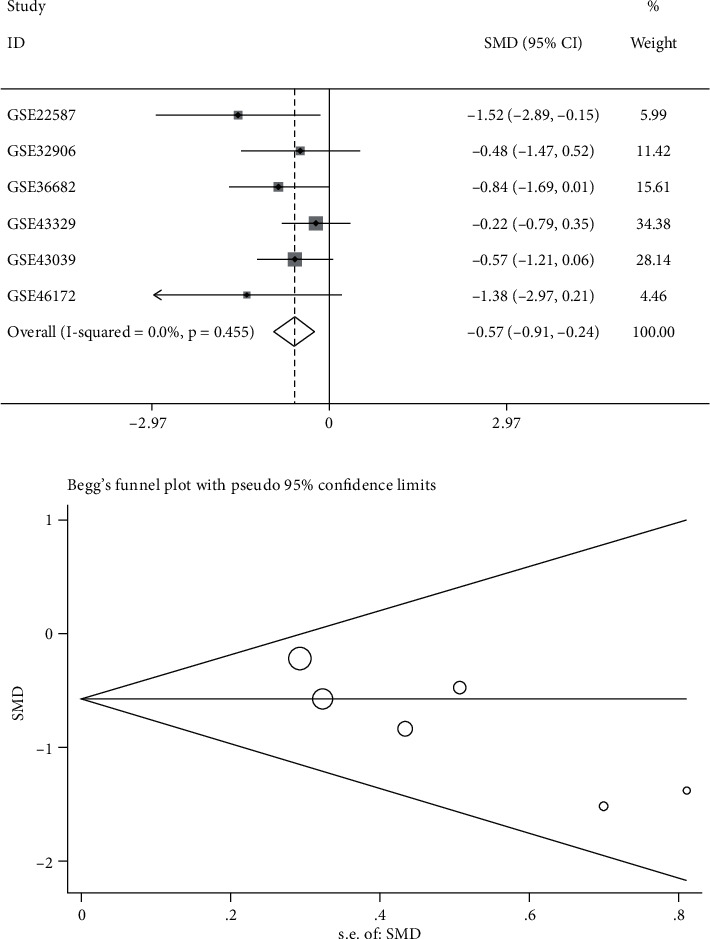
Pooled expression of miR-1 was calculated by a fixed effects model after excluding GSE32960: (a) forest plot and (b) publication bias.

**Figure 5:**
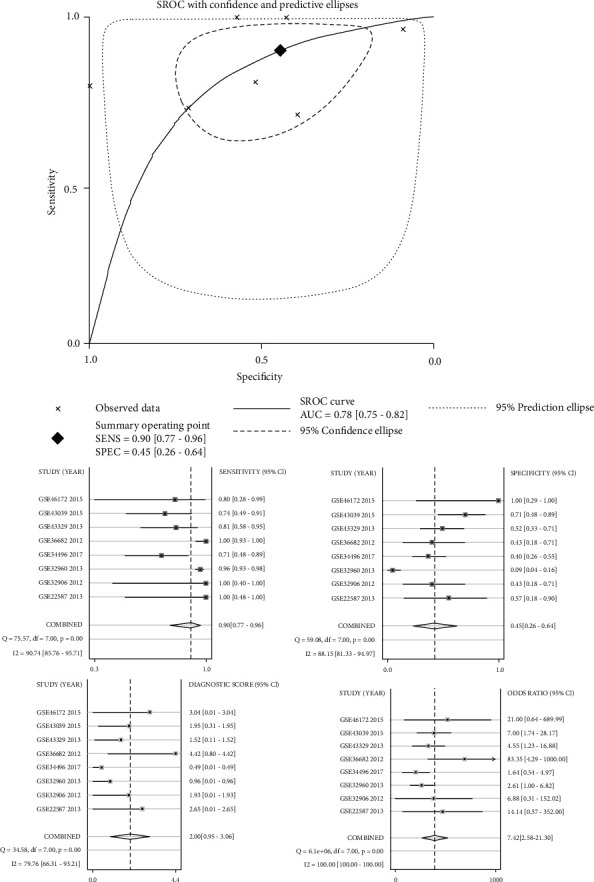
The ability of miR-1 to discriminate NPC from noncancerous tissues was measured by SROC.

To obtain reliable results, we integratively analyzed miR-1 expression in all miRNA microarray datasets. The results using the random effects model (*I*^2^ = 43.5%, *P* = 0.101; Figure 3(a)) showed that miR-1 expression was downregulated in NPC (SMD = −0.33; 95% CI: −0.61, −0.06; Figure 3(a)). Subsequently, we performed a sensitivity analysis (Figure 3(b)), which showed that the GSE32960 microarray dataset considerably affected the results. After excluding the GSE32960 microarray dataset, the pooled analysis result showed that miR-1 expression was still low in NPC (SMD = −0.57; 95% CI: −0.91, −0.24; Figure 4(a)).  Figure 4(b) suggests that all included datasets had no publication bias.  Figure 5 presents the SROC curve, with AUC of 0.78 (95% CI: 0.75–0.82) and sensitivity and specificity of 0.90 (95% CI: 0.77–0.96) and 0.45 (95% CI: 0.26~0.64), respectively, indicating that miR-1 had an excellent ability to distinguish cancerous tissues from noncancerous tissues.

### 3.2. Target Genes of miR-1

**Figure 6:**
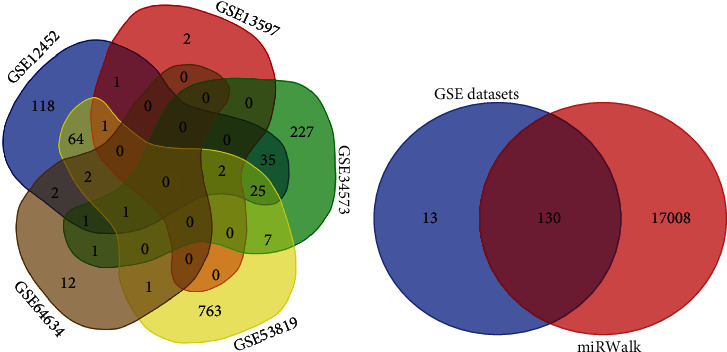
(a) shows the intersection of five GEO datasets. 143 genes were present in two or more datasets simultaneously. (b) shows the intersection of predicted target genes from miRWalk 2.0 and genes from (a).

As shown in Figure 6(a), 143 candidate target genes were obtained after the intersection of upregulated DEGs from five mRNA datasets of NPC. These 143 candidate target genes were then intersected with genes obtained from miRWalk 2.0, and finally, 130 potential target genes, such as cyclin-dependent kinase 1 (CDK1), ZNRF3, CDC45, CDCA2, and EZH2, were obtained for downstream analysis (Figure 6(b)).

### 3.3. Results of GO and KEGG Analysis

We utilized GO and KEGG analyses to identify the MFs, BPs, CCs, and cellular pathways that miR-1 participates in NPC.

**Figure 7:**
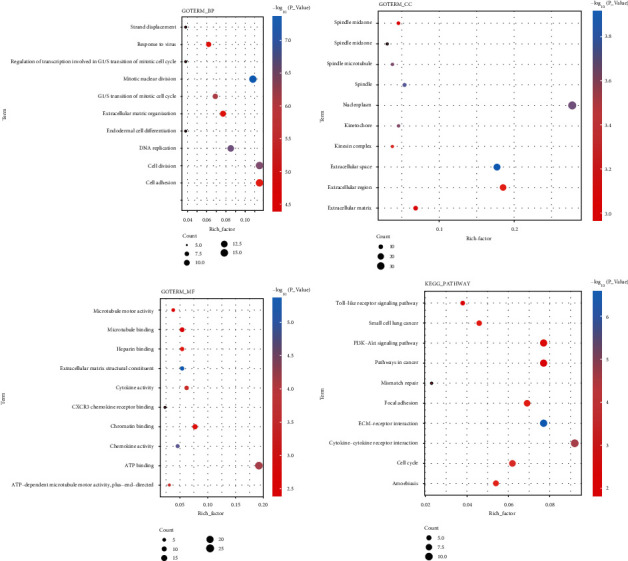
The top ten terms of GO and KEGG enrichment analysis of target genes.

For BP, the terms that are most significantly enriched in DNA replication, G1/S transition of mitotic cell cycle, cell division, cell adhesion, and mitotic nuclear division. In terms of CC, target genes mainly focused on extracellular space, kinesin complex, and extracellular matrix. As for MF, target genes were prominently enriched in extracellular matrix (ECM) structural constituent, microtubule motor activity, and microtubule binding. In addition, results of KEGG analysis suggested that target genes mainly focused on multiple cellular pathways, such as the PI3K/Akt signaling pathway, cell cycle, ECM-receptor interaction, focal adhesion, and cytokine-cytokine receptor interaction (Figure 7).

### 3.4. PPI Networks of Target Genes

**Figure 8:**
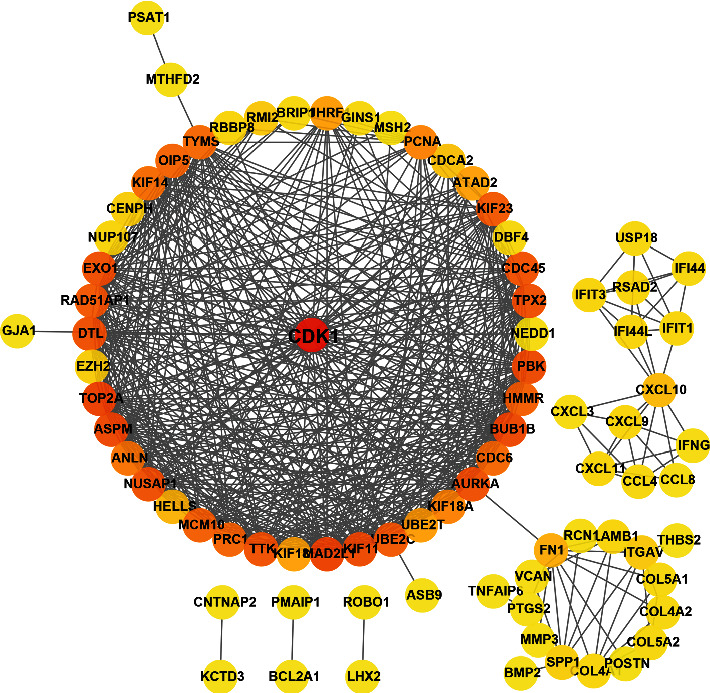
Protein-protein interaction network of 130 target genes was retrieved from the STRING database.

To further explore the interaction among target genes and determine the core genes in the regulatory network, we constructed a PPI network using the STRING database. CDK1 interacted with the largest number of genes (degrees ≥ 40) in the PPI network; thus, we considered CDK1 to be the key target gene (Figure 8).

### 3.5. Confirmation of CDK1 Expression by IHC

**Figure 9:**
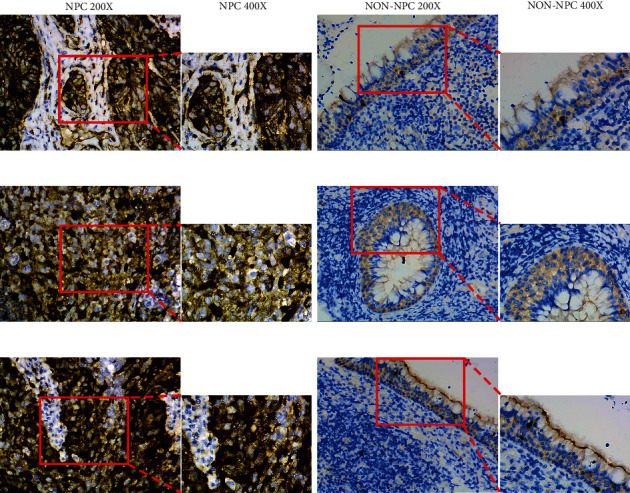
Expression of CDK1 in NPC tissues and noncancerous tissues was detected by immunohistochemistry.

CDK1 protein expression in thirty-six NPC tissues and twenty-eight nasopharyngeal mucosal chronic inflammation tissues was detected by IHC (Figure 9). An independent sample *t*-test of SPSS 26.0 was used to compare the IRS between the two groups, and the results indicated that the protein expression of CDK1 in NPC tissues (9.1389 ± 2.8998) was significantly higher than that in noncancerous tissues (0.7143 ± 1.0131, *P* < 0.05).

### 3.6. Molecular-Targeted Therapeutic Drugs for NPC

**Figure 10:**
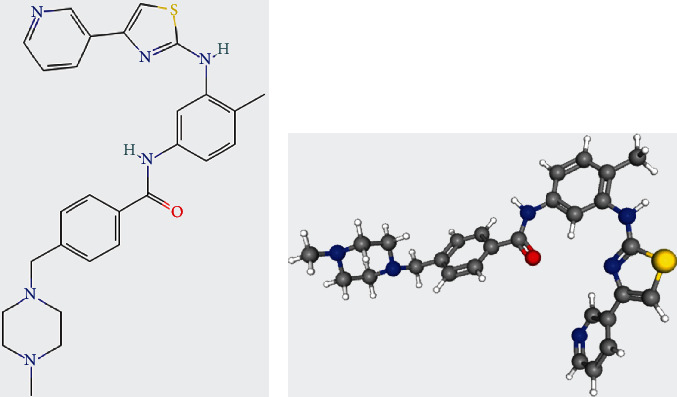
Schematic representation of the 2-dimensional (a) and 3-dimensional structure (b) of masitinib was derived from the PubChem database.

Eleven negatively correlated small-molecule compounds with the greatest association were screened using the cMap database, mainly including simvastatin, masitinib, droxinostat, mycophenolate-mofetil, and danusertib (Table 1). Among them, masitinib has attracted great interest as the only tyrosine kinase inhibitor (TKI). Masitinib, also known as AB1010, has a molecular formula of C_28_H_30_N_6_OS and a molecular weight of 498.6 g/mol.  Figure 10 shows the structure of masitinib. As a novel and elective TKI, it can not only block the activity of c-Kit receptor, fibroblast growth factor receptor 3, Lck/Yes-related protein, and lymphocyte-specific kinase, but it can also block the focal adhesion kinase (FAK) cell pathway by inhibiting FAK phosphorylation [30].

## 4. Discussion

Through the present study, we were the first to determine that miR-1 was targeted to regulate CDK1 in NPC, thereby affecting tumorigenesis and progression, and we further confirmed the upregulation of CDK1 by IHC. In addition, we further explored the key BPs, CCs, MFs, and pathways of miR-1 in NPC by GO analysis and KEGG pathway annotation. Finally, we confirmed that miR-1 led to NPC tumorigenesis and development by regulating ECM degradation, affecting the PI3K/Akt signaling pathway and participating in cancer cell mitosis.

Numerous studies have confirmed that miR-1 expression is decreased in various tumors; thus, miR-1 is considered a potential target for cancer therapy. In bladder cancer, miR-1 can induce G-S cell cycle arrest and inhibit cell proliferation by regulating Foxo1 and targeting Golgi phosphoprotein 3 [18]. miR-1 was downregulated in osteosarcoma. Overexpression of miR-1 can lead to increased p21 levels by targeting PAX3 and ultimately induce G0/G1 phase arrest and inhibit cell proliferation [17]. However, reports on the expression of miR-1 in NPC are scarce, and the mechanism has not been clarified.

In the present study, an overall analysis of seven miRNA microarray datasets derived from GEO, ArrayExpress, TCGA, and GTEx databases was conducted to clarify the low expression of miR-1 in NPC. A total of 130 target genes, including CDK1, ZNRF3, CDC45, CDCA2, and EZH2, were then screened by the comprehensive analysis of the predicted target genes from the miRWalk 2.0 database and the upregulated DEGs from the public database. Subsequently, the GO functional enrichment analysis of target genes showed that miR-1 was involved in NPC by regulating BPs, MFs, or CCs, such as mitotic nuclear division, DNA replication, cell division, cell adhesion, extracellular space, and kinesin complex. The results of the KEGG analysis suggested that the target genes mainly focused on multiple cellular pathways, such as PI3K/Akt signaling pathway, cell cycle, ECM-receptor interaction, focal adhesion, and cytokine-cytokine receptor interaction. For the PPI network, CDK1 was the most connected gene, and IHC confirmed the high CDK1 expression in NPC tissues. Finally, small-molecule compounds with potential for targeted treatment of NPC were screened by the cMap database.

The ECM consists of the basement membrane and intercellular substance and serves as a tissue barrier for carcinoma metastasis [31]. Carcinoma cells degrade the matrix by secreting or activating protein-degrading enzymes after adhesion of their surface receptors to many components in the ECM, forming a local lytic zone and constituting channels for metastasis; thus, tumor cells can metastasize to secondary sites and proliferate to form metastases [32, 33]. FAK is a nonreceptor tyrosine kinase that not only regulates cell development, growth, survival, and apoptosis but also regulates cell adhesion to ECM [34, 35]. FAK is overexpressed in various types of tumors and is associated with poor clinical prognosis [36, 37]. Machackova et al. demonstrated that miR-215-5p exerts a cancer-suppressive effect by affecting colorectal liver metastasis through regulating focal adhesion and ECM-receptor interaction [38]. Similarly, we suggest that miR-1 affects distant metastasis of NPC by regulating ECM and FAK. Besides, FAK is an upstream regulator of the PI3K/Akt pathway [39]. PI3K/Akt is a classical tumor signaling pathway [40]. Activation of this pathway can promote the proliferation of various tumor cells, including hepatocellular carcinoma, bladder cancer, and cervical carcinoma, inhibit tumor cell apoptosis, and resist sensitivity to radiotherapy and chemotherapy [41–43]. Liu et al. demonstrated that inhibition of the PI3K/Akt/NF-*κ*B signaling pathway reverses drug resistance in NPC [44]. Moreover, activation of the PI3K/Akt signaling pathway decreases the expression of the CDK inhibitors p21 and p27, thereby promoting cell cycle progression, which causes the proliferating cells to undergo uncontrollable proliferation by escaping from the regulation of p21 and p27, ultimately causing tumor progression [45–48]. CDK1, which is a member of the CDK family, plays a vital part in cell proliferation by regulating the G2/M transition in eukaryotic cell mitosis [49]. In this study, we confirmed the upregulation of CDK1 protein expression in NPC by IHC. Dysregulation of CDK1 was confirmed to cause cell cycle disturbance, which then leads to tumor production [50]. Numerous studies have reported that CDK1 is abnormally expressed in various tumors, such as melanoma, hepatocellular carcinoma, and pancreatic ductal adenocarcinoma, and is related to the degree of tumor malignancy [51–53]. Xie et al. demonstrated that CDK1 can regulate cell cycle progression, apoptosis, radioresistance, and cell growth of NPC [54]. Wang et al. demonstrated that triptolide can inhibit proliferation and induce apoptosis of NPC cells by regulating the PI3K/Akt pathway [55]. Hence, we conclude that miR-1 regulates p21 and p27 by affecting FAK and PI3K/Akt signaling pathways to affect CDK1 expression and ultimately lead to tumorigenesis. Masitinib, as a novel and effective tyrosine kinase inhibitor, can block the FAK cell pathway by inhibiting FAK phosphorylation and thus has the potential to be a targeted therapeutic agent for NPC [56].

In summary, our study concluded that miR-1 can regulate ECM adhesion function, activate the PI3K/Akt signaling pathway to inhibit p21/p27 expression, and lead to the upregulation of CDK1 by affecting FAK, ultimately prompting NPC tumorigenesis and progression. However, this conclusion, as well as the potential therapeutic ability of masitinib, must still be further verified by cell and animal experiments in future studies. Despite the limitations of our study, the results strongly suggest that miR-1 plays an important part in NPC and is expected to be a new therapeutic target.

## 5. Conclusion

This study was the first to determine that miR-1 may regulate ECM adhesion function, affect the PI3K/Akt signaling pathway, and participate in cancer cell mitosis by affecting FAK, thus playing an important part in NPC genesis, progression, and metastasis. This study will provide new therapeutic targets and insights for the treatment and tumor mechanism research of NPC in the future.

## Figures and Tables

**Table 1:** The top eleven negatively correlated small-molecule compounds from the cMap database. These small-molecule compounds have the potential to be the targeted therapeutic agents for NPC.

Score	ID	Name	Description
-99.79	BRD-A81772229	Simvastatin	HMGCR inhibitor
-99.72	BRD-K71035033	Masitinib	KIT inhibitor
-99.19	BRD-K11558771	Droxinostat	HDAC inhibitor
-99.08	BRD-K92428153	Mycophenolate-mofetil	Dehydrogenase inhibitor
-98.94	BRD-K07881437	Danusertib	Aurora kinase inhibitor
-98.88	BRD-K35430135	SR-59230A	Adrenergic receptor antagonist
-98.45	BRD-K57080016	Selumetinib	MEK inhibitor
-98.01	BRD-K51313569	Palbociclib	CDK inhibitor
-97.89	BRD-K00615600	AG-14361	PARP inhibitor
-97.85	BRD-K50836978	Purvalanol-a	CDK inhibitor
-97.71	BRD-K13049116	BMS-754807	IGF-1 inhibitor

## Data Availability

miRNA and mRNA expression data of this study were derived from open access public databases. Immunohistochemistry results are available from the corresponding author upon reasonable request.
